# Gait decline while dual-tasking is an early sign of white matter deterioration in middle-aged and older adults

**DOI:** 10.3389/fnagi.2022.934241

**Published:** 2022-09-29

**Authors:** Haidar Alzaid, Thomas Ethofer, Bernd Kardatzki, Michael Erb, Klaus Scheffler, Daniela Berg, Walter Maetzler, Markus A. Hobert

**Affiliations:** ^1^Department of Biomedical Magnetic Resonance, Tübingen University Hospital, Tübingen, Germany; ^2^Department of Neuroradiology, Heidelberg University Hospital, Heidelberg, Germany; ^3^Department of Psychiatry and Psychotherapy, Tübingen University Hospital, Tübingen, Germany; ^4^Department of Neurology, Kiel University Hospital, Kiel, Germany

**Keywords:** diffusion tensor imaging, dual-task costs, gait, white matter, executive functions

## Abstract

Loss of white matter integrity (WMI) is associated with gait deficits in middle-aged and older adults. However, these deficits are often only apparent under cognitively demanding situations, such as walking and simultaneously performing a secondary cognitive task. Moreover, evidence suggests that declining executive functions (EF) are linked to gait decline, and their co-occurrence may point to a common underlying pathology, i.e., degeneration of shared brain regions. In this study, we applied diffusion tensor imaging (DTI) and a standardized gait assessment under single- and dual-tasking (DT) conditions (walking and subtracting) in 74 middle-aged and older adults without any significant gait or cognitive impairments to detect subtle WM alterations associated with gait decline under DT conditions. Additionally, the Trail Making Test (TMT) was used to assess EF, classify participants into three groups based on their performance, and examine a possible interaction between gait, EF, and WMI. Gait speed and subtracting speed while dual-tasking correlated significantly with the fractional anisotropy (FA) in the bilateral anterior corona radiata (highest *r* = 0.51/*p* < 0.0125 FWE-corrected). Dual-task costs (DTC) of gait speed correlated significantly with FA in widespread pathways, including the corpus callosum, bilateral anterior and superior corona radiata, as well as the left superior longitudinal fasciculus (highest *r* = −0.47/*p* < 0.0125 FWE-corrected). EF performance was associated with FA in the left anterior corona radiata (*p* < 0.05); however, EF did not significantly mediate the effects of WMI on DTC of gait speed. There were no significant correlations between TMT and DTC of gait and subtracting speed, respectively. Our findings indicate that gait decline under DT conditions is associated with widespread WM deterioration even in middle-aged and older adults without any significant gait or cognitive impairments. However, this relationship was not mediated by EF.

## Introduction

Gait impairments are common with aging and lead to major consequences, including falls, disability, and long-term institutionalization (Scheffer et al., 2008). However, many gait impairments are not apparent and are presumably masked by compensatory strategies. For instance, some gait impairments are only apparent in cognitively demanding situations, such as under cognitive-motor dual-tasking (DT) conditions (Lord et al., 2013). This is especially true in older people with executive function (EF) impairments, as executive dysfunction has been reported to be associated with slower gait under DT conditions and could be a possible mediator of falls (Yogev-Seligmann et al., 2008). In fact, the decline in EF appears to be an early sign of global cognitive decline and frailty. It is reasonable to assume a link between gait and cognition, as this has been consistently demonstrated in studies (Yogev-Seligmann et al., 2008; Montero-Odasso et al., 2009; Hobert et al., 2017); however, the mechanism behind it is not entirely clear. It has been theorized that gait in complex situations requires recruiting additional (primarily cognitive) resources (Boisgontier et al., 2013). In this regard, gait decline under DT conditions could reflect deterioration of shared white matter (WM) pathways, which are also involved in EF.

Previous evidence suggests the involvement of mostly frontal WM pathways in gait under DT conditions, including the corpus callosum (Ghanavati et al., 2018; Castro-Chavira et al., 2019; Snir et al., 2019), the anterior corona radiata (ACR)/anterior thalamic radiation (Pettit et al., 2013; Ruggieri et al., 2018; Castro-Chavira et al., 2019; Snir et al., 2019), and the left superior corona radiata (Hupfeld et al., 2022). However, this relationship has been mostly investigated in patient groups and older people with dementia. As frontal WM is particularly vulnerable to normal aging (Pfefferbaum et al., 2005), it is important to test this relationship in non-demented adults to assess the utility of gait assessment under DT conditions as an early indicator of WM deterioration. Moreover, further investigations into this relationship are needed, including the possible role of executive functions as a mediator and the existence of shared pathways between motor and cognitive tasks.

In this study, we applied Diffusion Tensor Imaging (DTI) to detect such alterations in the aging population and determine their influence on EF and gait under DT conditions. DTI is a modern neuroimaging method with a high sensitivity to detect WM alterations (Basser et al., 1994), which has been successfully applied to identify the WM correlates of gait (Bhadelia et al., 2009) and cognitive-motor dual-tasking (Snir et al., 2019) in older adults. Using this imaging method, researchers can obtain DTI-derived parameters to examine WM Integrity (WMI), such as fractional anisotropy (FA), which reflects diffusion orientation and coherence along the fibers. Low FA values generally indicate reduced WMI (Pierpaoli and Basser, 1996).

By assessing gait and cognitive function with a DT paradigm at the behavioral level and WMI using DTI, we examined whether gait decline under DT conditions is associated with early WM alterations in middle-aged and older adults without any significant gait or cognitive impairments. We then classified the participants into three groups based on their EF performance and compared WMI between the groups. Subsequently, a mediation analysis was conducted to assess whether EF mediates the interaction between WMI and gait under DT conditions. We hypothesize that the deterioration of widespread white matter contributes to gait decline while dual-tasking. Additionally, deterioration of shared pathways between the motor and cognitive tasks and pathways involved in executive functions might influence this interaction.

## Materials and methods

### Participants

This is a cross-sectional study as a part of the TREND study (Tübinger evaluation of Risk factors for Early Detection of Neurodegeneration), a prospective biannual follow-up study aimed at defining biomarkers for early detection of Parkinson’s disease and Alzheimer’s disease (Gaenslen et al., 2014). For this MRI cross-sectional study, we recruited 74 older adults from the whole cohort of the TREND study (41 women, mean age: 67 ± 6 years). All participants were middle-aged and older adults between 55 and 81 years of age and met the criteria of being community-dwelling without any significant gait or cognitive impairments. Specifically, all participants could walk without ambulatory aids or assistance, had a minimum score of 24 on the Mini-Mental State Examination (MMSE) (Folstein et al., 1975), and had no clinical signs of dementia. They also had no diagnosis of any neurodegenerative disease, a history of stroke, epilepsy, central nervous system inflammatory diseases, or polyneuropathy. They were free of antipsychotic or other antidopaminergic drugs and exhibited no significant impairment of vision or hearing. All participants gave written informed consent, and the study was approved by the ethical committee of the University of Tübingen (Nr. 90/2009BO2 and 370/2013BO2).

### Trail making test

All participants underwent testing of EF based on parts A and B of the TMT (Reitan and Skills, 1958). In part A, participants have to connect numbers from 1 to 25 using a pen. It is used to assess visual search ability and motor speed. In part B, subjects have to connect numbers and letters alternatingly (1-A-2-B-3-C….). In addition to the components from part A, part B also assesses cognitive flexibility and working memory. We chose the derived score B-A as our parameter of interest, as previous studies have recommended using the derived score B-A to minimize the influence of motor speed and visuospatial perception and provide a more reliable indicator of EF (Sanchez-Cubillo et al., 2009). The participants were categorized based on their TMT B-A performance into three groups. TMT B-A values > 54 s defined poor performers (*n* = 17), values ranging from 35 to 54 s defined intermediate performers (*n* = 25), and values < 35 s defined good performers (*n* = 32). The cut-off values used are from a related analysis from the TREND study with a much larger and more representative sample size (*N* = 1054) (Salkovic et al., 2017).

### Single- and dual-task assessment

In the single-task (ST) and DT assessments, the participants performed two ST and one DT trials. In the motor ST trial, participants were instructed to walk as fast as possible for 20 m on at least a 1.5-m wide corridor. Participants in the cognitive ST trial subtracted serial 7s as fast as possible from a random three-digit number until ten subtractions were made; the task was performed while standing. The time for subtracting was measured with a stopwatch, and the examiner took the number of subtractions in DT. The subtracting speed was defined as the number of subtractions divided by the time needed for the task (seconds).

In the DT trial, participants simultaneously performed the subtracting the walking tasks. Both tasks had to be performed as fast as possible without any hints given by the investigator regarding the prioritization of any task. The time for walking and subtracting was measured with a stopwatch, and the examiner took the number of subtractions in DT.

Performing the trials at a fast walking pace as opposed to a preferred walking pace is meant to increase their difficulty level and reveal gait deficits masked by compensatory cognitive control of gait (Lord et al., 2013). There was only one dual-task trial to measure the “natural performance” without a chance to improve/change the strategy. For the familiarization, the two tasks were performed in a single task condition.

While there is no standardized protocol for testing gait under ST and DT conditions, the reliability of our protocol has been established in previous studies (Hobert et al., 2011, 2017; Salkovic et al., 2017).

To test the individual DT ability regardless of their ST performance, we derived the relative DT parameters, dual-task costs (DTC) of gait and subtracting speed, using the following formula (Baddeley et al., 1997).

DTC[%]=[(STspeed-DTspeed)/STspeed]*100

Dual-task costs measure the change in DT compared to ST performance, expressed as a percentage of ST performance. Positive DTC describe a deterioration in DT, and negative DTC describe an improvement in DT compared to ST.

We decided to also include the absolute DT parameters in the analysis as an exploratory measure, as they could provide additional information needed to fully understand DT mechanisms (Plummer and Eskes, 2015).

We thus derived the following clinical parameters for the dual-task assessment:

(1) DT gait speed while subtracting.
(2) DT subtracting speed while walking.
(3) DTC of gait speed (while subtracting).
(4) DTC of subtracting speed (while walking).

### Image acquisition

Structural T1-weighted images (TR = 2300 ms, TE = 4.18 ms, TI = 900 ms, voxel size: 1 × 1 × 1 mm^3^) were acquired with a 3 Tesla scanner (Siemens PRISMA, Erlangen, Germany). Diffusion-weighted images were acquired using a “Stejskal-Tanner” sequence (TR = 6.0 s, TE = 69 ms, flip angle = 90°, 50 axial slices, 2 acquisitions) with a voxel size of 1.7 × 1.7 × 2.5 mm^3^ along 30 independent directions using a *b*-Value of 1000 s/mm^2^. Additionally, 12 images with a *b*-Value of 0 s/mm^2^ were acquired throughout the sequence. The clinical tests and image acquisition were done in separate sessions.

### Diffusion tensor imaging data analysis

Data preprocessing and DTI analysis were carried out using FSL 5.0.9 (FMRIB Software Library^1^). Preprocessing included correction of eddy current distortions and motion artifacts (Andersson and Sotiropoulos, 2016). Head motion was compared between groups across six directions. The DTIFIT tool was used to fit a diffusion tensor model at each voxel and generate the FA map. A voxel-wise statistical analysis of the FA data was carried out using the Tract-Based Spatial Statistics (TBSS) included in FSL (Smith et al., 2004, 2006). The FA maps from each subject were aligned into a common space using the nonlinear registration tool FNIRT (Andersson et al., 2007), which uses a b-spline representation of the registration warp field (Rueckert et al., 1999), and finally averaged to create a mean FA image. The mean FA image was then thinned to create a mean FA skeleton, representing the centers of all tracts common to the group; an FA threshold of 0.2 was used for the skeleton. Each subject’s aligned FA map was then projected onto this skeleton. The correlation between FA and the aforementioned dual-tasking parameters was set up using the GLM tool in FSL and included age and gender as covariates of no interest. Using the “Randomise” tool incorporated in FSL, we ran nonparametric permutation-based statistical tests on the DTI maps with 10,000 permutations and threshold-free cluster enhancement (Smith and Nichols, 2009) to correct for multiple comparisons across the whole brain at a significance threshold of *p* < 0.05 using family-wise error correction; the *p*-Value threshold was further reduced to *p* < 0.0125 (*p* = 0.05 divided by 4, Bonferroni corrected for four DT parameters). We then extracted the significant clusters within each tract based on Johns Hopkins University’s Mori WM atlas to compare group FA values (Mori et al., 2008). The significant maps of DT gait speed and DT subtracting speed were multiplied to determine areas of overlap.

### Statistical analysis

Statistical analysis of clinical and neuropsychological measures was carried out using SPSS version 25 software (SPSS Inc., Chicago, Illinois). A one-way ANCOVA (age and gender as covariates of no interest) and the *post hoc* Tukey-HSD or the Kruskal−Wallis and *post hoc* Dunn’s tests were used to compare mean scores between the groups for normally and non-normally distributed parameters, respectively. The X^2^ test was used to assess potential differences in gender distribution and the prevalence of relevant comorbidities. A non-corrected *p*-Value of <0.05 was considered significant for all clinical and neuropsychological tests. We compared FA values between the three groups in the clusters that showed a significant correlation with DTC of gait speed using a one-way ANCOVA adjusted for age, gender, and hypertension, followed up by *post hoc* analysis using Tukey’s HSD test to assess pairwise differences while correcting for multiple comparisons. Group comparisons were only carried out in significant clusters of >30 voxels (6 ANCOVAs). Using PROCESS macro v.3.4.1 implemented in SPSS^2^, we used the simple mediation model (model 4) to assess whether TMT B-A performance (i.e., EF) mediates the effects of FA (only in clusters with a significant group difference) on DTC of gait speed. The mediation was calculated using 95% confidence intervals with a bootstrapping sample size of 5,000. Age, gender, and hypertension were included as covariates of no interest.

Additionally, we ran a linear correlation analysis (Pearson) between TMT B-A and DTC of gait speed and subtracting speed. We performed a partial correlation analysis of the four DT measures adjusted for age and gender for descriptive purposes. A *p*-Value of <0.0125 was considered significant (*p* = 0.05 divided by 4, Bonferroni corrected for four DT parameters).

## Results

### Demographic and clinical features

Gender distribution and years of education, as well as MMSE, were not significantly different between the groups (Table 1). There was also no significant difference in the prevalence of comorbidities. The poor TMT group was significantly older than the good TMT group (*p* < 0.05) and also older than the intermediate TMT group. However, the latter comparison did not reach statistical significance. ST subtracting speed significantly differed between good and poor performers, but all other ST and DT parameters and DTC scores did not. There were no significant correlations between TMT B-A and DTC of gait (*p* = 0.1, *r* = 0.18) and subtracting speed (*p* = 0.15, *r* = −0.17), respectively. There was a significant correlation between the four DT measures (Table 2).

**TABLE 1:** Demographic and clinical parameters of the groups.

	Whole cohort (SD)	Good TMT (SD)	Intermediate TMT (SD)	Poor TMT (SD)	*p*
N (female)	74 (41)	32 (20)	25 (12)	17 (9)	0.53
Age (years)	66.8 (6.5)	65.5 (5.4)^a^	65.9 (7.0)	70.4 (6.6)	<0.05
Education (years)	14.6 (2.6)	15.1 (2.8)	13.8 (2.0)	14.8 (2.6)	0.14
MMSE (0−30)	28.7 (1.4)	28.8 (1.4)	28.5 (1.5)	28.7 (1.5)	0.64
Osteoarthritis (%)	51.4	46.9	52.0	58.8	0.73
Osteoporosis (%)	5.4	9.4	0	5.9	0.29
Obesity (%)	14.9	18.8	16.0	5.9	0.47
Hypertension (%)	37.8	25.0	44.0	52.9	0.12
Diabetes (%)	6.8	9.4	8.0	0	0.44
TMT A (s)	34.5 (10.1)	33.4 (9.9)	32.8 (9.5)	39.2 (10.5)	0.23
TMT B (s)	76.0 (26.6)	56.3 (12.5)^b^	75.8 (10.2)^c^	113.4 (22.6)	<0.0001
TMT B-A (s)	41.5 (22.9)	22.9 (8.1)^b^	43.0 (6.3)^c^	74.1 (19.4)	<0.0001
ST gait speed (m/s)	1.67 (0.21)	1.64 (0.23)	1.72 (0.22)	1.66 (0.12)	0.30
ST subtracting speed (1/s)	0.34 (0.14)	0.39 (0.09)^d^	0.30 (0.16)	0.29 (0.13)	<0.005
DT gait speed while subtracting (m/s)	1.34 (0.20)	1.32 (0.19)	1.38 (0.22)	1.29 (0.19)	0.53
DT subtracting speed while walking (1/s)	0.29 (0.14)	0.32 (0.13)	0.26 (0.17)	0.28 (0.12)	0.14
DTC of gait speed while subtracting (%)	19.5 (12.0)	18.2 (12.8)	19.9 (11.2)	21.6 (12.0)	0.56
DTC of subtracting speed while walking (%)	12.0 (33.3)	15.6 (28.5)	15.2 (42.9)	0.84 (24.3)	0.26

Group comparisons were performed using a one-way ANCOVA and the *post hoc* Tukey-HSD test or the Kruskal−Wallis and the *post hoc* Dunn’s test for normally and non-normally distributed parameters, respectively. SD, standard deviation; MMSE, Mini-Mental State Examination; ns, not significant, TMT, trail making test; DT, dual-task; DTC, dual-task costs.

^*a*^Significant difference (*p* < 0.05) compared to the poor TMT group.

^*b*^Significant difference (*p* < 0.0001) compared to the intermediate and poor TMT groups.

^*c*^Significant difference (*p* < 0.005) compared to the poor TMT group.

^*d*^Significant difference (*p* < 0.005) compared to the intermediate and poor TMT groups.

**TABLE 2:** Partial correlation between the dual-tasking parameters adjusted for age and gender.

Parameter	DT gait speed	DT subtracting speed	DTC of gait speed	DTC of subtracting speed
DT gait speed	−	0.29/0.013	−0.62/0.00001	−0.37/0.001
DT subtracting speed	0.29/0.013	−	−0.36/0.002	−0.56/0.00001
DTC of gait speed	−0.62/0.00001	−0.36/0.002	−	0.36/0.002
DTC of subtracting speed	−0.37/0.001	−0.56/0.00001	0.36/0.002	−

The values are expressed as *r/p*.

DT, dual-task; DTC, dual-task costs.

### Tract-based spatial statistics

**FIGURE 1:**
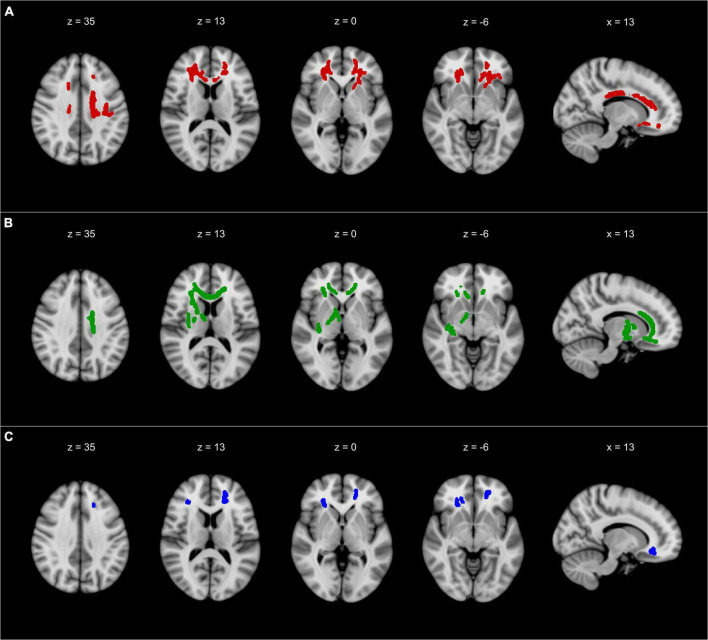
The results of TBSS whole-brain correlation analysis overlaid on MNI-152 T1 (1 mm) template and presented in radiological convention. **(A)** Negative correlation between FA and DTC of gait speed while subtracting. **(B)** Positive correlation between FA and DT gait speed while subtracting. **(C)** Positive correlation between FA and DT subtracting speed while walking. All TBSS results were thickened to improve visualization and are presented with a threshold of *p* < 0.0125 corrected for multiple comparisons. TBSS, tract-based spatial statistics; FA, fractional anisotropy; DT, dual-task; DTC, dual-task costs; x, x-coordinate; z, z-coordinate. Detailed description of the significant clusters, including cluster sizes and correlation coefficients, can be found in Table 3.

**FIGURE 2:**
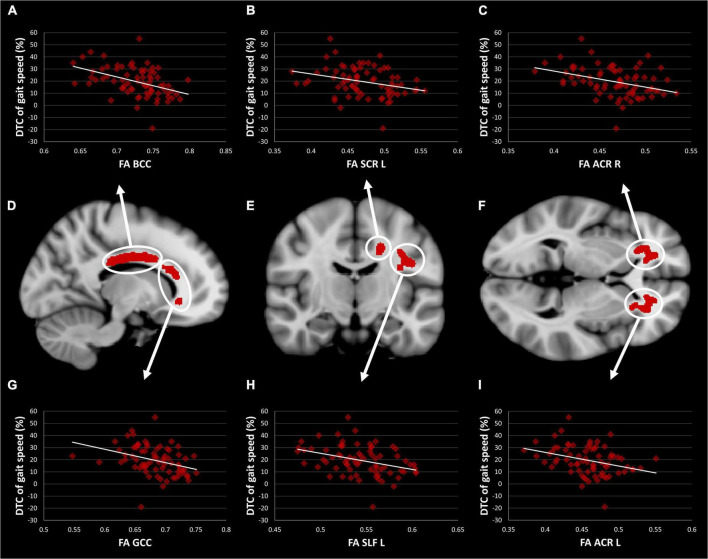
TBSS Clusters with a significant correlation between DTC of gait speed while subtracting and FA **(D–F)** displayed on T1 MNI-152 template (1 mm). Scatter plots showing a correlation between FA and DTC of gait speed while subtracting in body **(A)** and genu **(G)** of the corpus callosum, left superior corona radiata **(B)**, left superior longitudinal fasciculus **(H)**, right **(C)** and left **(I)** anterior corona radiata. TBSS, tract-based spatial statistics; FA, fractional anisotropy; DTC, dual-task costs; GCC, genu of the corpus callosum; BCC, body of the corpus callosum; ACR, anterior corona radiata; SCR, superior corona radiata; SLF, superior longitudinal fasciculus.

The TBSS-based whole-brain correlation analysis revealed a significant correlation between FA and three dual-tasking parameters (positive correlation with DT subtracting speed while walking, positive correlation with DT gait speed while subtracting, and negative correlation with DTC of gait speed while subtracting) in several WM regions (see Figures 1, 2). Significant clusters with a voxel size of >5 are presented in Table 3. There was no significant correlation between FA and DTC of subtracting speed while walking using a threshold of *p* < 0.0125 corrected for multiple comparisons. There was an overlap between the correlation maps of DT gait speed and DT subtracting speed in the bilateral ACR (left ACR 88 voxels, right ACR 104 voxels).

**TABLE 3:** Group differences in clusters with a significant partial correlation between dual-task parameters and FA adjusting for age, gender, and hypertension.

Parameter	Region	Cluster size (voxels)	*r*/*p*	FA				*p*
				Whole cohort	Good TMT	Intermediate TMT	Poor TMT	
DTC of gait speed while subtracting	GCC	309	−0.40/0.0006	0.683 ± 0.004	0.692 ± 0.005	0.681 ± 0.007	0.667 ± 0.012	0.188
	BCC	1073	−0.47/0.00003	0.727 ± 0.004	0.736 ± 0.005	0.724 ± 0.008	0.715 ± 0.009	0.184
	ACR L	631	−0.41/0.0003	0.458 ± 0.004	0.473 ± 0.005	0.451 ± 0.008	0.442 ± 0.008	0.008*
	ACR R	693	−0.44/0.0001	0.466 ± 0.004	0.475 ± 0.005	0.462 ± 0.008	0.457 ± 0.008	0.308
	SCR L	267	−0.36/0.002	0.471 ± 0.005	0.484 ± 0.005	0.467 ± 0.009	0.454 ± 0.009	0.096
	SLF L	190	−0.39/0.0008	0.543 ± 0.004	0.552 ± 0.005	0.535 ± 0.007	0.539 ± 0.008	0.162
	SCC	29	−0.36/0.01	0.843 ± 0.004	0.845 ± 0.005	0.838 ± 0.007	0.846 ± 0.011	0.697
	ALIC L	18	−0.28/0.02	0.468 ± 0.007	0.475 ± 0.012	0.472 ± 0.012	0.450 ± 0.017	0.546
	CG L	12	−0.35/0.003	0.628 ± 0.007	0.631 ± 0.012	0.619 ± 0.011	0.635 ± 0.013	0.359
	EC L	15	−0.28/0.02	0.481 ± 0.004	0.480 ± 0.007	0.482 ± 0.007	0.482 ± 0.010	0.587
	EC R	23	−0.41/0.0004	0.450 ± 0.004	0.450 ± 0.006	0.450 ± 0.006	0.454 ± 0.009	0.338
	SCR R	13	−0.20/0.09	0.444 ± 0.007	0.460 ± 0.009	0.434 ± 0.012	0.430 ± 0.015	0.186
DT gait speed while subtracting	GCC	864	0.47/0.00004	0.725 ± 0.004	0.730 ± 0.005	0.727 ± 0.006	0.715 ± 0.010	0.480
	BCC	823	0.46/0.00006	0.727 ± 0.004	0.734 ± 0.005	0.726 ± 0.009	0.713 ± 0.009	0.306
	ACR L	364	0.37/0.002	0.483 ± 0.005	0.497 ± 0.006	0.477 ± 0.009	0.467 ± 0.011	0.065
	ACR R	699	0.46/0.00005	0.500 ± 0.004	0.503 ± 0.005	0.501 ± 0.007	0.492 ± 0.007	0.875
	SCR L	151	0.36/0.002	0.501 ± 0.005	0.511 ± 0.006	0.498 ± 0.010	0.483 ± 0.009	0.257
	ALIC R	213	0.38/0.001	0.645 ± 0.004	0.640 ± 0.006	0.650 ± 0.006	0.647 ± 0.008	0.627
	EC R	247	0.45/0.00007	0.504 ± 0.003	0.514 ± 0.004	0.495 ± 0.006	0.497 ± 0.007	0.021
	RIC R	26	0.30/0.01	0.601 ± 0.004	0.608 ± 0.007	0.598 ± 0.008	0.589 ± 0.009	0.493
	SCR R	352	0.46/0.00006	0.575 ± 0.003	0.583 ± 0.004	0.566 ± 0.005	0.573 ± 0.007	0.022
	PLIC R	272	0.37/0.001	0.690 ± 0.003	0.690 ± 0.005	0.688 ± 0.006	0.695 ± 0.007	0.811
	SS R	14	0.25/0.04	0.574 ± 0.006	0.585 ± 0.009	0.559 ± 0.011	0.574 ± 0.011	0.139
	FST R	39	0.30/0.01	0.608 ± 0.006	0.612 ± 0.007	0.619 ± 0.011	0.584 ± 0.010	0.274
	CG L	43	0.35/0.003	0.634 ± 0.006	0.638 ± 0.009	0.632 ± 0.011	0.631 ± 0.012	0.551
DT subtracting speed while walking	ACR L	174	0.44/0.0001	0.438 ± 0.005	0.447 ± 0.007	0.435 ± 0.009	0.424 ± 0.010	0.354
	ACR R	166	0.51/0.00001	0.514 ± 0.004	0.521 ± 0.004	0.512 ± 0.009	0.504 ± 0.009	0.488

All values are expressed as the mean ± standard error. FA, fractional anisotropy; TMT, trail making test; DT, dual-task; DTC, dual-task costs; GCC, genu of the corpus callosum; BCC, body of the corpus callosum; SCC, splenium of the corpus callosum; ACR, anterior corona radiata; SCR, superior corona radiata; SLF, superior longitudinal fasciculus; ALIC, anterior limb of the internal capsule; RIC, retroarticular part of the internal capsule; PLIC, posterior limb of the internal capsule; CG, cingulum (cingulate gyrus); EC, external capsule; SS, sagittal stratum; FST, fornix/stria terminalis. Only clusters with a voxel size > 5 are presented. *Significantly higher FA value in the good TMT group compared to the intermediate (*p* < 0.05) and poor TMT groups (*p* < 0.01).

### Group comparisons

**FIGURE 3:**
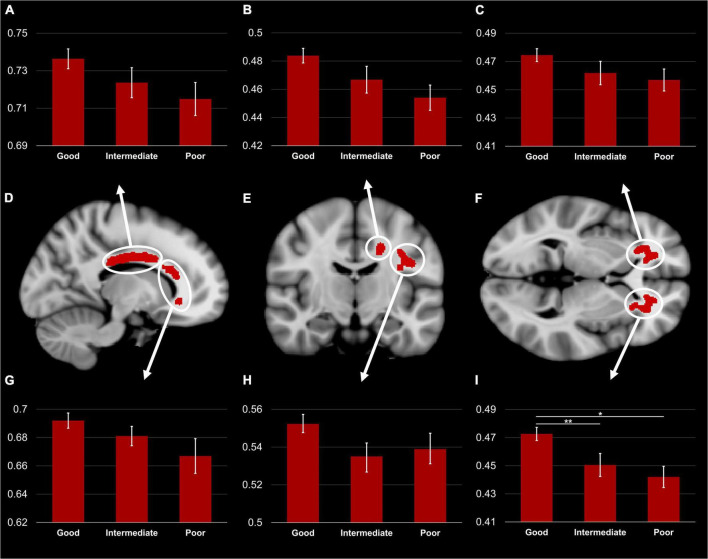
TBSS Clusters with a significant correlation between DTC of gait speed while subtracting and FA **(D–F)** displayed on T1 MNI-152 template (1 mm). Group comparison of FA in body **(A)** and genu **(G)** of the corpus callosum, left superior corona radiata **(B)**, left superior longitudinal fasciculus **(H)**, right **(C)** and left **(I)** anterior corona radiata. Error bars represent standard error of the mean. TBSS, tract-based spatial statistics; FA, fractional anisotropy; DTC, dual-task costs. *Significant difference between the good and poor TMT groups (*p* < 0.01). **Significant difference between the good and intermediate TMT groups (*p* < 0.05).

The one-way ANCOVA revealed a significant difference in FA values between the three TMT groups in the left ACR [*F*(2,71) = 5,14; *p* < 0.05, Bonferroni corrected for six clusters, see Figure 3]. The other five clusters showed a similar pattern, but the ANCOVAs obtained no significant results [all *F*(2,71) < 2.2; all *p* > 0.05]. Within the left ACR, the good TMT group had a significantly higher FA value than the intermediate TMT (*p* < 0.05) and poor TMT groups (*p* < 0.01). The FA value was higher in the intermediate TMT group compared to the poor TMT group, but this comparison failed to reach statistical significance. The mediation analysis was used to determine whether executive functions (TMT B-A performance) mediated the effects of FA in the left ACR on DTC of gait speed. However, the result was not significant (β = −0.0271, SE = 0.0485, 95% confidence interval [−0.1197, 0.0781]).

## Discussion

We investigated the association between WMI and gait decline under DT conditions in middle-aged and older adults, and explored the possible role of EF in this interaction. The key findings of our study are that (i) gait decline while dual-tasking was associated with loss of WMI in frontoparietal pathways, including the corpus callosum, (ii) the bilateral ACR appears to be a shared pathway for the cognitive and motor tasks, and (iii) EF performance was associated with WMI in the left ACR; however, EF did not mediate the interaction between WMI and gait decline under DT conditions.

We demonstrated that DTC of gait speed was negatively correlated with FA predominantly in frontoparietal pathways, including the corona radiata, the corpus callosum, the superior longitudinal fasciculus, and the internal and external capsule. This finding suggests that gait decline under DT conditions is associated with widespread microstructural changes in cerebral WM in middle-aged and older adults. Previous DTI studies demonstrated similar findings in patients with amyotrophic lateral sclerosis (Pettit et al., 2013) and multiple sclerosis (Ruggieri et al., 2018), as well as individuals with mild cognitive impairment (Snir et al., 2019). WMI in these pathways is also associated with habitual gait speed in healthy older adults (Poole et al., 2018).

The strongest correlations with FA were found in the corpus callosum and anterior corona radiata. The corpus callosum is a hub for interhemispheric communication to relay motor and cognitive information (Van Der Knaap and Van Der Ham, 2011). Evidence from earlier studies suggests that WMI in the corpus callosum is essential for maintaining gait under ST (Bhadelia et al., 2009; Poole et al., 2018; Snir et al., 2019) and DT conditions (Pierpaoli and Basser, 1996), in addition to its important role in EF (Halliday et al., 2019). Similarly, the ACR is a pathway known for its crucial role in maintaining adequate DT performance (Pettit et al., 2013; Ruggieri et al., 2018; Snir et al., 2019). Furthermore, the ACR is also involved in key components of EF, i.e., working memory and cognitive flexibility (Schmahmann et al., 2009; Macpherson and Hikida, 2019). This pathway is a part of the basal ganglia-thalamocortical circuitry connecting the prefrontal cortex (PFC) and the anterior cingulate cortex with subcortical structures, such as the thalamus and the basal ganglia. Interestingly, a recent paper revealed a positive association between DTC of gait speed and gray matter volume in the PFC and the cingulate cortex in a cohort of 139 healthy older adults (mean age = 75 years) (Tripathi et al., 2019). The authors interpreted their findings as an increased importance of these regions as DT performance worsens, e.g., as a compensatory mechanism. Another study investigating 55 healthy older adults (mean age = 75 years) showed that lower gray matter volume in frontal areas leads to over-activation of the frontal lobe during a cognitively demanding walking task, which supports the notion of neural inefficiency in aging (Wagshul et al., 2019). Numerous studies measured brain activity using different techniques and reported increased brain activity in these regions for gait under DT conditions compared to ST conditions (Leone et al., 2017). The increased neural activity also compensates for prefrontal WM deterioration, i.e., less wiring and more firing (Daselaar et al., 2015; Lucas et al., 2019). With respect to these studies, our findings highlight the importance of frontal regions in maintaining gait under DT conditions. However, DTC of gait speed might be more sensitive to WM alterations, as gray matter areas can functionally and/or structurally compensate for changes in DTC of gait speed.

Corresponding to the results of the DTC of gait speed, our exploratory analysis of the absolute DT parameters revealed a significant correlation between FA in predominantly frontal WM and the absolute parameters of gait speed and subtracting speed in DT conditions. Interestingly, the WM correlates of both the cognitive and the motor tasks in DT conditions overlapped in the ACR (Figures 1B,C), thus indicating that both the motor and the cognitive tasks share WM pathways. Some authors suggest that gait under challenging conditions requires the recruitment of additional mainly cognitive brain regions, most notably the PFC, which becomes increasingly difficult with aging (Boisgontier et al., 2013). As previously mentioned, the ACR represents the primary connections of the PFC, thereby concurring with previous findings. However, some pathways correlated significantly with DT gait speed but not with DT subtracting speed, including the corpus callosum and the internal and external capsule. In light of these findings, gait decline under DT conditions might only partially be explained by the deterioration of shared WM pathways.

Our group comparison demonstrated a markedly lower FA in the left ACR in the poor and intermediate TMT groups compared to the good TMT group. Similar patterns were observed in the other WM regions associated with increased DTC of gait speed; however, they did not reach statistical significance. This result concurs with a previous study demonstrating an association between damage to left-lateralized frontal areas and EF deficits on four neuropsychological tests (including TMT) (Barbey et al., 2012).

While it may be intriguing to assume a direct relationship between low EF and increased DTC of gait speed based on shared substrates, the correlation analysis we ran between TMT B-A and DTC of gait speed revealed an insignificant and weak positive correlation (*p* = 0.1, *r* = 0.18). Furthermore, our mediation analysis did not reveal any significant results. In other words, TMT B-A performance did not mediate the effects of FA on DTC of gait speed.

A similar imaging study revealed a significant correlation between TMT B-A and normal gait speed but not with the DTC of gait speed. However, the authors reported an association between DTC of gait speed and episodic memory performance, implying that ST and DT gait are supported by different brain networks (Tripathi et al., 2019). However, this finding has not been replicated and contradicts the findings of many clinical studies. It is also plausible that these inconsistent results are due to the relatively small sample size in MRI studies, as a related clinical study from TREND with a much larger sample size (*N* = 661) revealed a highly significant and slightly stronger correlation between TMT B-A and DT gait speed while subtracting (*p* < 0.0001, *r* = 0.27) (Hobert et al., 2017). In addition to the well-recognized critical role of EF in complex gait situations, e.g., walking over an obstacle course (Ble et al., 2005) or walking under DT conditions (Yogev-Seligmann et al., 2008; Hobert et al., 2017). Nevertheless, EF is only one factor affecting a complex multifactorial mechanism such as gait (Jayakody et al., 2018), especially under DT conditions, which could also explain the relatively weak correlation in our study.

It is worth mentioning that despite the apparent difference in FA values across the groups in clusters associated with DTC of gait speed, only a slight gradual increase in the DTC value between the good TMT (18%), intermediate TMT (20%), and poor TMT groups (22%) was observed, although it was not statistically significant. While this may contradict one of the most cited papers regarding the association between EF and gait in complex situations (Ble et al., 2005), the authors used much higher TMT B-A thresholds in older subjects (mean age = 75 years); some of whom have global cognitive impairment. As a result, we conclude that an association between DTC of gait speed and EF might only be apparent with a high degree of EF impairments and/or global cognitive impairments.

Interestingly, two recent studies provide a plausible explanation for this phenomenon, as they have shown that compromised WM microstructural integrity (Lucas et al., 2019) and reduced frontal gray matter volume (Wagshul et al., 2019) were accompanied by an increase in oxygenated hemoglobin in the PFC during dual-task walking. This could be able to, at least partly, compensate for the increasing cognitive demands of locomotion and perhaps decrease gait deterioration under DT conditions. It is worth mentioning that some studies have found an association between DTC and other cognitive functions, namely episodic (Montero-Odasso et al., 2014) and working memory (Montero-Odasso et al., 2009), suggesting the potential involvement of temporal regions. Accordingly, future studies should investigate the structural correlates of DTC and their association with multiple cognitive domains. In summary, this result suggests that EF and gait performance in DT conditions share common neural substrates. However, the effect of WM alterations on DT gait impairments might be compensated by other mechanisms in the early stages of cognitive impairments.

Even though our cohort did not have any significant gait impairments, their gait performance declined under the more challenging DT conditions, as measured by DTC. Moreover, gait decline was associated with a lower FA value, i.e., lower WMI, in widespread WM pathways. Our findings thus suggest that the decrement of gait performance under DT conditions could be an early sign of WM deterioration. In this regard, testing DTC of gait might be of clinical importance in the aging population, as it provides opportunities for early therapeutic, preventive, and behavioral interventions.

This study has some limitations. For example, the study did not include T2-FLAIR images to estimate the burden of microvascular associated WM changes, i.e., WM hyperintensities, which can influence DTI measures such as FA (Vangberg et al., 2019), and have also been associated with gait impairments (Bolandzadeh et al., 2014), and executive dysfunction (Murray et al., 2010) in community-dwelling older adults. Nevertheless, our cohort exhibits a relatively low cardiovascular burden, corresponding to normal aging in this age group.

Furthermore, we assessed the effects of only one secondary cognitive task on gait impairments under dual-tasking. Existing evidence suggests that different cognitive tasks involving internal interfering factors (e.g., mental tracking and verbal fluency tasks) disturb gait and posture equally, regardless of their nature (Bloem et al., 2003; Al-Yahya et al., 2011). A DTI study revealed distinct WM correlates of gait speed under dual-tasking conditions depending on the nature of the paired cognitive task (subtracting 7s, counting, naming animals) (Snir et al., 2019). This could, however, be a consequence of using a fairly unspecific parameter that does not take into account task prioritization, i.e., “walking speed while dual-tasking,” instead of the more specific parameter we applied here: DTC of gait speed. Additionally, we only evaluated the gait parameter gait speed and did not consider DTC changes related to other gait parameters. While other gait parameters, such as stride length and cadence, are also susceptible to dual-task-related changes, gait speed appears to be the most sensitive parameter to these changes and also the most utilized gait parameter in DT studies (Al-Yahya et al., 2011).

## Conclusion

Our findings indicate that gait decline under DT conditions is an early sign of WM deterioration, even in middle-aged and older adults without any significant gait or cognitive impairments. Some of the affected WM pathways were shared between the cognitive and motor tasks, indicating that gait decline under DT conditions might be partially explained by the deterioration of shared WM pathways. However, this relationship was not mediated by EF.

## Data availability statement

The original contributions presented in this study are included in the article/supplementary material, further inquiries can be directed to the corresponding author.

## Ethics statement

The studies involving human participants were reviewed and approved by the Ethical Committee of the University of Tübingen (Nr. 90/2009BO2 and 370/2013BO2). The patients/participants provided their written informed consent to participate in this study.

## Author contributions

HA, MH, and TE contributed to the conception and design of the study. DB, KS, TE, MH, and WM organized the study. HA performed data analysis and wrote the first draft. BK and ME provided analysis tools. MH and TE wrote sections of the manuscript. All authors contributed to the manuscript revision, read, and approved the submitted version.

## Footnotes

1
http://fsl.fmrib.ox.ac.uk/fsl/

2
www.processmacro.org
